# Plasma Deposition and UV Light Induced Surface Grafting Polymerization of NIPAAm on Stainless Steel for Enhancing Corrosion Resistance and Its Drug Delivery Property

**DOI:** 10.3390/polym10091009

**Published:** 2018-09-10

**Authors:** Ko-Shao Chen, Shu-Ju Chang, Chi-Kuang Feng, Win-Li Lin, Shu-Chuan Liao

**Affiliations:** 1 Department of Materials Engineering, Tatung University, Taipei 104, Taiwan; kschen@ttu.edu.tw (K.-S.C.); smy0215@gmail.com (S.-J.C.); 2 Department of Orthopaedics and Traumatology, Taipei Veterans General Hospital, Taipei 112, Taiwan; frankckfeng@gmail.com; 3 Institute of Biomedical Engineering, National Taiwan University, Taipei 106, Taiwan; winli@ntu.edu.tw; 4 Bachelor Program for Design and Materials for Medical Equipment and Devices, Da Yeh University, Changhua 515, Taiwan

**Keywords:** plasma deposition, ultraviolet (UV) light, surface modification, hydrogels, drug delivery

## Abstract

When stainless steel is implanted in human bodies, the corrosion resistance and biocompatibility must be considered. In this study, first, a protective organic silicone film was coated on the surface of stainless steel by a plasma deposition technique with a precursor of hexamethyldisilazane (HMDSZ). Then, ultraviolet (UV) light-induced graft polymerization of *N*-isopropylacrylamide (NIPAAm) and acrylic acid (AAc) in different molar ratios were applied onto the organic silicone film in order to immobilize thermos-/pH-sensitive composite hydrogels on the surface. The thermo-/pH-sensitive composite hydrogels were tested at pH values of 4, 7.4 and 10 of a phosphate buffer saline (PBS) solution at a fixed temperature of 37 °C to observe the swelling ratio and drug delivery properties of caffeine which served as a drug delivery substance. According to the results of Fourier Transformation Infrared (FTIR) spectra and a potential polarization dynamic test, the silicone thin film formed by plasma deposition not only improved the adhesion ability between the substrate and hydrogels but also exhibited a high corrosion resistance. Furthermore, the composite hydrogels have an excellent release ratio of up to 90% of the absorbed amount after 8h at a pH of 10. In addition, the results of potential polarization dynamic tests showed that the corrosion resistance of stainless steel could be improved by the HMDSZ plasma deposition.

## 1. Introduction

A hydrogel is a three-dimensional network hydrophilic polymer that features in water-absorbing and swelling phenomena [1,2]. The hydrogel can be contracted or expanded by its response to chemical or physical transitions such as temperature, pressure, and pH value, etc. Hydrogels have applications in the human body [3,4,5].

Cold plasma treatment has been found to be a unique surface modification technology. The films produced by plasma activation treatment have a good adhesion and coverage [6,7]. A uniform organic film with low porosity can be produced by this method regardless of whether the substrate is conductive or not. Moreover, the peroxides resulting from the residual free radicals can be further modified by a light grafting polymerization technique to allow the substrate surface to create a special active radicals structure for biomedical applications such as the immobilization of antibodies and DNA, and drug detection, etc. [8,9]. Such a technique is performed under vacuum and only requires surface modification without altering the bulk properties of the material.

Plasma enhanced chemical vapor deposition (PECVD) together with a ultraviolet (UV) light graft polymerization process can create bonds between the substrate and the organic biomedical molecules. The UV light graft polymerization process can achieve the purpose of selectively grafting and cross-linking monomers of different functional groups, and its features include the simplicity of its process, diversity, and a low associated pollution [10,11]. In order to increase the polymer adhesion to the substrate surface, surface roughness plays an important role [12,13]. The surface roughness possesses directionality and can effectively improve the polymerization and adhesion of substrates like acrylic acid straight-chain monomers. The roughening surface has a greater surface area and facilitates the adhesion of more polymers. In addition, the holes on the roughening surface are favorable for the adhesion of polymer, causing the deposited film to be securely in place and unlikely to peel off [14,15,16].

Poly(*N*-isopropylacrylamide) (PNIPAAm) is a special kind of polyelectrolyte, which contains carbonyl groups and amide groups along with a long and hydrophobic backbone chain. In aqueous solution, slightly crosslinked PNIPAAm forms a soft gel that has a reversible volume phase transition in response to changes in temperature [17]. This transition has substantial importance from medical, technological, and scientific points of view. In the past decade, PNIPAAm has approached body temperature due to its high biocompatibility and temperature sensitivity (LCST = 32 °C), it has been extensively studied to become the most widely used smart polymer with uses in various applications over a wide range of fields, including materials, biomedical, drug delivery system drugs, etc. [18,19].

In this study, a cold plasma deposition system was first used to clean the stainless steel specimens by introducing argon gas into the reaction chamber. The hexamethyldisilazane (HMDSZ) was then introduced into the reaction chamber to start the plasma deposition treatment under different conditions to form an anti-corrosion film on the specimens. After the plasma deposition, UV induced graft polymerization of *N*-isopropylacrylamide (NIPAAm) and acrylic acid (AAc) in different molar ratios was carried out to immobilize a thermo-/pH-sensitive composite hydrogel on the specimen surface. A potential dynamic polarization test was conducted to determine the corrosion resistance of the specimen. The specimens subjected to various treatments were characterized by water contact angle, Fourier Transformation Infrared (FTIR), Scanning Electron Microscope (SEM), and Electron Spectroscopy for Chemical Analysis (ESCA). The thermo-/pH-sensitive composite hydrogels were tested at pH values of 4, 7.4 and 10 of phosphate buffer saline (PBS) solution and at a temperature of 37 °C to observe its swelling ratio and drug delivery properties.

## 2. Materials and Methods

### 2.1. Pretreatment of Stainless Steel

AISI 304 stainless steel sheets with a thickness of 1 mm were cut into pieces of 15 mm × 12 mm as substrates. The substrate surface was blasted and then successively washed with alcohol and deionized water ultrasonically to remove dirt and residual fine sands.

### 2.2. Cold Plasma Treatment

**Figure 1:**
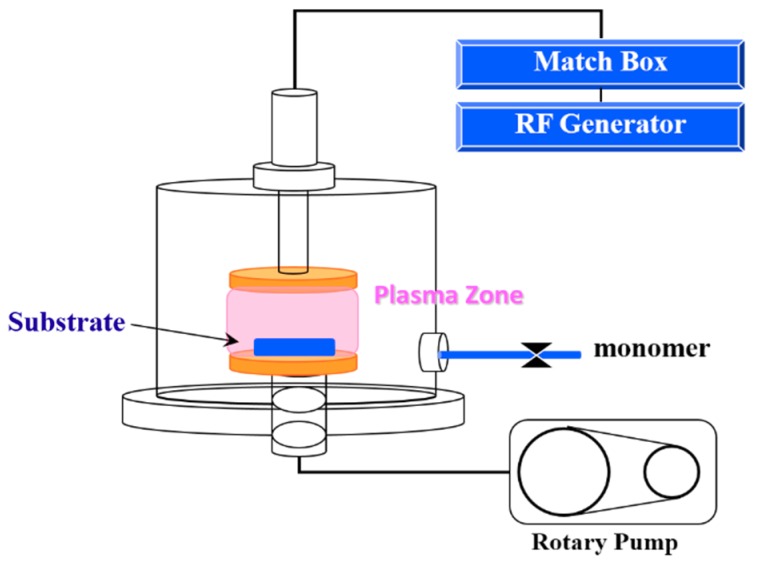
Schematic diagram of the plasma treatment system.

Figure 1 shows the plasma polymerization equipment with a bell-jar reaction chamber and 13.56 MHz radio frequency generators (model PD-2S manufactured by SAMCO Co., Kyoto, Japan). A vacuum pump was employed to provide a low-pressure environment. The cleaned stainless steel specimens were placed on the lower electrode of the reaction chamber before being evacuated. The reaction chamber was evacuated to less than 30 mTorr. The argon gas was introduced into the reaction chamber and maintained at a constant pressure by adjusting the micro-throttle valve in the argon-inlet tube. After the pressure was stabilized, the specimen surface was cleaned at an input power of 30 W using argon gas of 100 mTorr for 1 min. In order to obtain an organic hydrophobic film on the stainless steel surface, the surface must be coated with HMDSZ by a cold plasma process. The monomers HMDSZ were purchased from Fluka Chemical Co., New York, NY, USA, ((CH_3_)_3_Si–NH–Si(CH_3_)_3_), Mw = 161.39 g/mol). The reactor chamber pressure was controlled by gas input and vacuum pump throttle valves through which HMDSZ monomers were introduced into the plasma chamber at a pressure of 60 mTorr. As the gas input is controlled by monomer evaporation, exact calculation of the flow rate is not possible. The processing power was at either 20 or 50 W for treatment times of 3, 10, 20 and 30 min, respectively.

### 2.3. Composite Hydrogels by Ultraviolet (UV) Light Surface Graft Polymerization

The HMDSZ-treated stainless steel specimen was soaked in an aqueous solution of mixed NIPAAm(H_2_C = CHCONHCH(CH_3_)_2_, Mw = 113.16 g/mol) and AAc (CH_2_ = CHCOOH, Mw = 72.06 g/mol) monomer solution and vitamin B_2_ with a ratio of monomer solution to B_2_ of 4:1. The aqueous solution also contains 5 mol % of *N*’-methylene-bis-acrylamide (NMBA, (H_2_C = CHCONH)_2_CH_2_, Mw = 154.17 g/mol), 1 mol % of Ammonium Persulfate(APS, (NH_4_)_2_S_2_O_8_, Mw = 228.20 g/mol), and 1 mol % of *N*,*N*,*N*′,*N*′-tetramethylethylenediamine (TEMED, (CH_3_)_2_NCH_2_CH_2_N(CH_3_)_2_, Mw = 116.20 g/mol) with different molar ratios of NIPAAm and AAc monomers. The compositions of the Poly (NIPAAm/AAc) copolymer hydrogels were listed in Table 1. Graft polymerization was performed under UV light (power of 1000 W and wavelength of 365 nm) exposure for 5 min. After graft polymerization, the grafted specimens were washed with distilled water overnight to remove the homopolymer aqueous solution.

### 2.4. Surface Characterization

Surface morphology of the samples was observed using a scanning electron microscope (JSM 5600, JEQL, Tokyo, Japan). The Fourier transform infrared spectrometer (Jasco FT/IR-300E, Jasco, Tokyo, Japan) was used to analyze the surface functional groups after the plasma modification, UV light graft polymerization, and immobilization of composite hydrogels. The chemical compositions of the modified surfaces were measured by ESCA. The grafting density and the swelling rate were also measured.

### 2.5. Potential Dynamic Polarization Test

The potential dynamic polarization tests were performed using a Solartron 1287 electrochemical apparatus (Solartron Analytical, Farnborough, UK) in a 3.5% NaCl solution at room temperature. A standard three-electrode system was employed. The Ag/AgCl electrode was used as the reference electrode, and the platinum plate as the counter electrode. All potentials were expressed with respect to the reference electrode. The potential scanning rate was controlled at 1 mV/s.

### 2.6. Drug Release Test

Caffeine was used as a drug in the drug release test. The dry gels were equilibrated in a solution of 30 mg of the drug and 1 L of deionized water (30 ppm) at 25 °C for 3 days. The drug delivery experiments were carried out by transferring previously incubated drug gels into a 10 mL phosphate buffer solution at 37 °C. The gels were repeatedly removed and transferred into a fresh 10 mL buffer solution at each fixed time interval. The drug release amount was determined by using UV-Vis (JASCO V530) to measure the transmittance and then converted to the release amount.

## 3. Results and Discussion

### 3.1. Scanning Electron Microscope (SEM) Morphology

**Figure 2:**
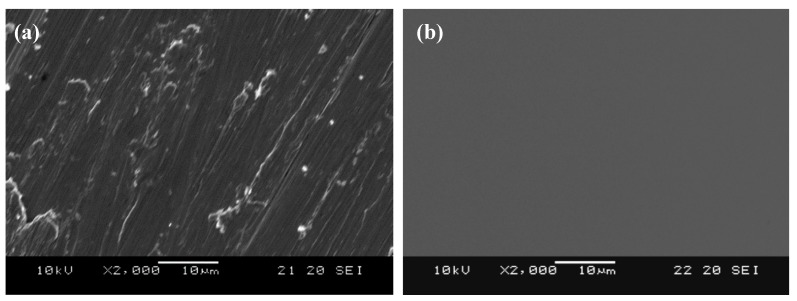
Surface morphologies of (**a**) un-modified and (**b**) hexamethyldisilazane (HMDSZ)-plasma treated (at 20 W for 20 min) for 304 stainless steel specimens.

**Figure 3:**
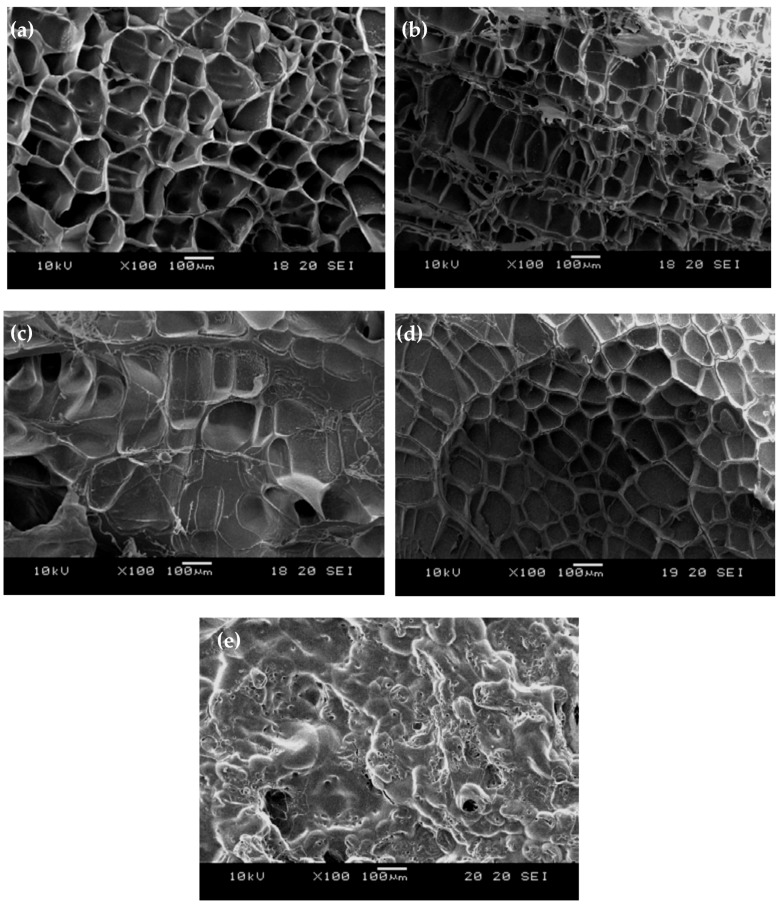
Scanning Electron Microscope (SEM) micrographs of freeze-dried stainless steel specimens subjected to HMDSZ plasma treatment and grafting with Poly (NIPAAm/AAc) composite hydrogels under different molar ratios of AAc. (**a**) NIPAAm (0 mol % AAc), (**b**) 1 mol % AAc, (**c**) 3 mol % AAc, (**d**) 5 mol % AAc, and (**e**) 10 mol % AAc.

Figure 2 shows the surface morphologies of (a) un-modified and (b) HMDSZ-treated (at 20 W for 20 min) for 304 stainless steel specimens. The surface of un-modified stainless steel appears very rough, and after the treatment the surface becomes smooth. The dense HMDSZ films deposited on the substrate surface contribute to the enhancement of the corrosion resistance. Figure 3a–e shows the freeze-dried surface network SEM micrographs of stainless steel specimens subjected to HMDSZ plasma treatment and grafting with Poly (NIPAAm/AAc) composite hydrogels under different molar ratios of AAc. The pore sizes shown in Figure 3a–d were 100.0 ± 5.6, 86.8 ± 13.7, 80.4 ± 2.3, and 70.88 ± 3.1 μm, respectively. The figure indicated that as the AAc concentration increases, the number of pores is increased and the pore size is decreased. In addition, the pore depth becomes shallower.

### 3.2. Fourier Transformation Infrared (FTIR) Characterization of Structure Evolution

**Figure 4:**
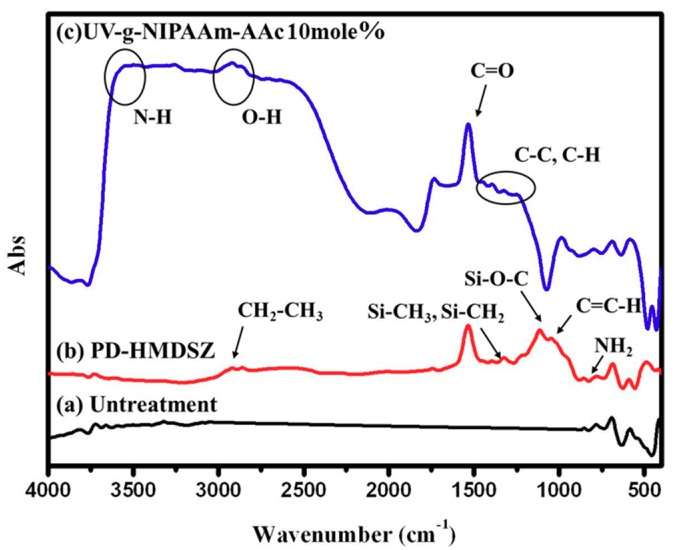
Fourier Transformation Infrared (FTIR) spectra of (**a**) un-modified, (**b**) HMDSZ-treated, and (**c**) NIPAAm/AAc hydrogel-grafted stainless steel specimens.

Figure 4 shows the FTIR spectra of (a) un-modified, (b) HMDSZ-treated (at 60 mtorr and 20 W for 20 min), and (c) NIPAAm/AAc hydrogel-grafted (with NIPAAm/10 mol % AAc) stainless steel specimens. No functional group was found in the un-modified stainless steel specimen because of its inorganic nature. The spectrum in Figure 4b revealed the presence of a C–H stretch of methyl groups at 2800 cm^−1^ and 3000 cm^−1^, Si–N–Si at 900–1180 cm^−1^, SiC at 1180–1260 cm^−1^, and SiCH_3_ at 770−875 cm^−1^. The functional group N–H peak of NIPAAm was found at 3200~3600 cm^−1^, O–H peak was found at 3000 cm^−1^, C–H was found at 1424 cm^−1^, and C=O peak was found at 1700–1720 cm^−1^. As for the AAc, its functional group C=O peak appears at 1727 cm^−1^, the –C–O–C– peak at 1261 cm^−1^, and the COH peak at 1095 cm^−1^. Both NIPAAm and AAc have the C=O peak in common, but the difference in both is the O–H peak [13,14,15]. As the AAc concentration in the NIPAAm/AAc hydrogel increases, the peaks pertaining to the AAc functional groups become stronger and the NIPAAm functional groups become weaker.

### 3.3. Chemical Composition Analysis

**Figure 5:**
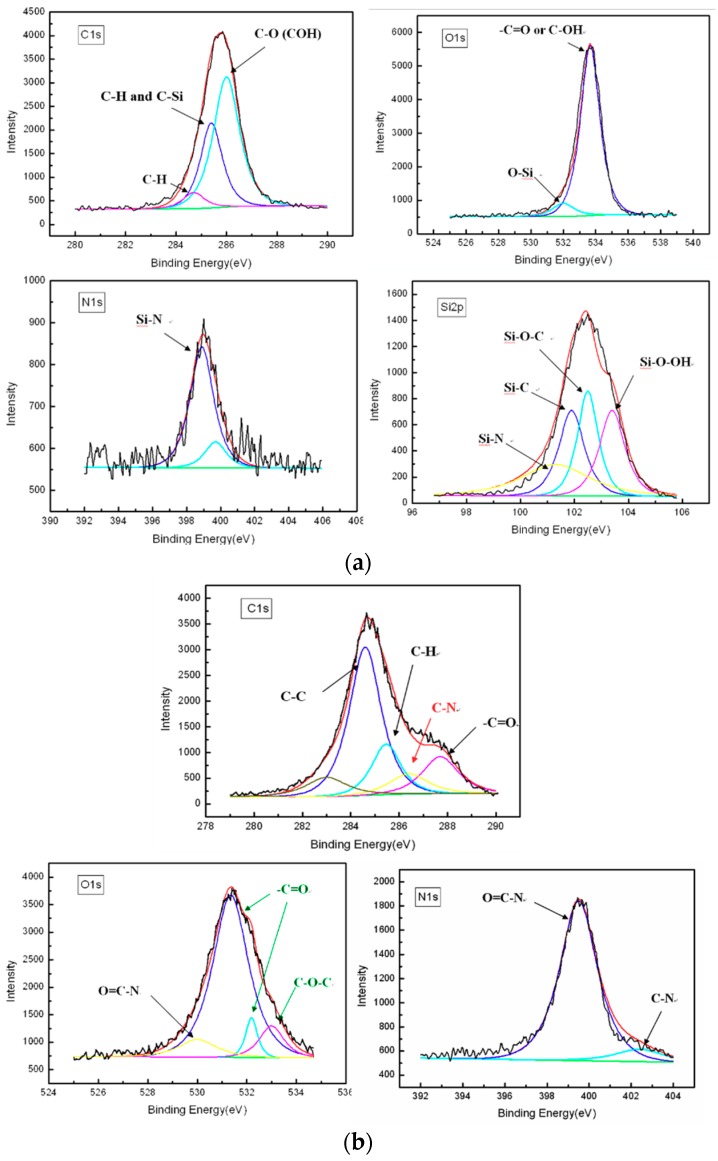
Electron spectroscopy for chemical analysis (ESCA) spectra of (**a**) HMDSZ-treated (20 W, 20 min, 60 mtorr), and (**b**) Poly (NIPAAm/10 mol % AAc) composite hydrogels grafted onto a HMDSZ-treated substrate.

ESCA analysis is used to characterize the chemical bonding and functional groups of the surface film. In this study, a Si wafer was used as the substrate for ESCA analysis. After the plasma treatment the peaks of Si2p, C1s, O1s, and N1s, which are the constituent elements of HMDSZ, were detected on the specimen surface, as shown in Figure 5a. The Si2p ESCA spectrum of the surface film can be fitted by five peaks, each representing a separate Si bond; Si–N (100.2 eV), Si–C (102.9 eV), Si–O–C (102.3 eV), and Si–O–OH (103.5 eV), The C1s ESCA spectrum of the surface films can be fitted by three peaks, each representing a separate C bond; C–H (284.7 eV), C–H,C–Si (285.4 eV), and CO (COH) (286.9 ev), The O1s ESCA spectrum of the surface films can be fitted by two peaks, each representing a separate O bond; O–Si (532.0 eV), and –C=O or C–OHC–H (533.65 eV), The N1s ESCA spectrum of the surface films can be fitted by one peaks is Si–N (398.9 eV). The chemical compositions of the grafted Poly (NIPAAm/AAc) composite hydrogel were also measured by the ESCA analysis; Figure 5b shows that the C1s ESCA spectrum of Poly(PNIPAAm/10 mol % AAc) composite hydrogels grafted onto the surface of HMDSZ-treated substrate can be fitted by four peaks, C–C (284.3 eV), C–H (285.2 eV), C–N (286.3 eV), and C=O (287.6 eV), respectively, the O1s can be fitted by three peaks, O=C–N (530.0 eV), –C=O (532.2 eV) and C–O–C (533.0 eV),the N1s can be the fitted by two peaks, O=C–N (399.5 eV) and C–N (402.4 eV). Peak component C–N was associated with the carbon atom attached directly to the nitrogen atom in the amino group. It was attest that Poly (NIPAAm/AAc) hydrogels grafted onto the substrate successful. Table 2 lists the atomic concentration of Poly (NIPAAm/AAc) hydrogels grafted onto the HMDSZ-treated substrate.

### 3.4. Wettability of the Modified Surface

**Figure 6:**
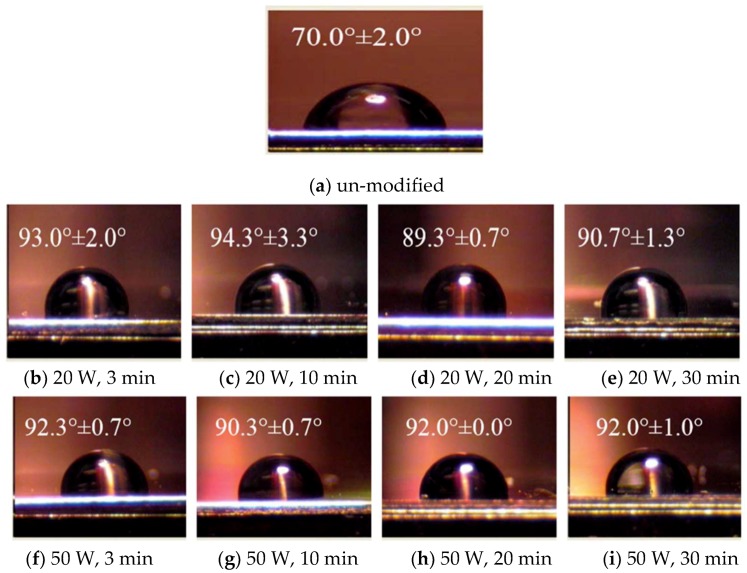
The water contact angle of HMDSZ plasma deposited (PD-HMDSZ) stainless steel (PD-HMDSZ: 60 mTorr). (**a**) un-modified, (**b**) 20 W, 3 min, (**c**) 20 W, 10 min, (**d**) 20 W, 20 min, (**e**)20 W, 3 min, (**f**) 50 W, 3 min, (**g**) 50 W, 10 min, (**h**) 50 W, 20 min and (**i**) 50 W, 3 min.

The water contact angles (WCA) on the un-modified and the plasma-deposited specimens were measured by the sessile drop method with distilled water at room temperature. The data were recorded by a CCD camera (Goni-meter type G-1, ERMA Optical Works, Tokyo, Japan). Each WCA value was the average of four measurements. A large contact angle reveals that the surface is more hydrophobic. The measured water contact angle of un-modified stainless steel is 70.0 ± 2.0°. Figure 6 shows the water contact angle of HMDSZ-deposited stainless steel after plasma deposition of HMDSZ at an input power of 20 W and 50 W for different durations, showing that the surface exhibits a hydrophobic tendency and is covered with a membrane of organic silicone and alkyl-like compounds. The WCA values seem to change little with the increase in the plasma treatment time, and short time HMDSZ plasma treatment is preferable to form a hydrophobic protected film.

### 3.5. Corrosion Resistance from Potential Dynamic Polarization Test

**Figure 7:**
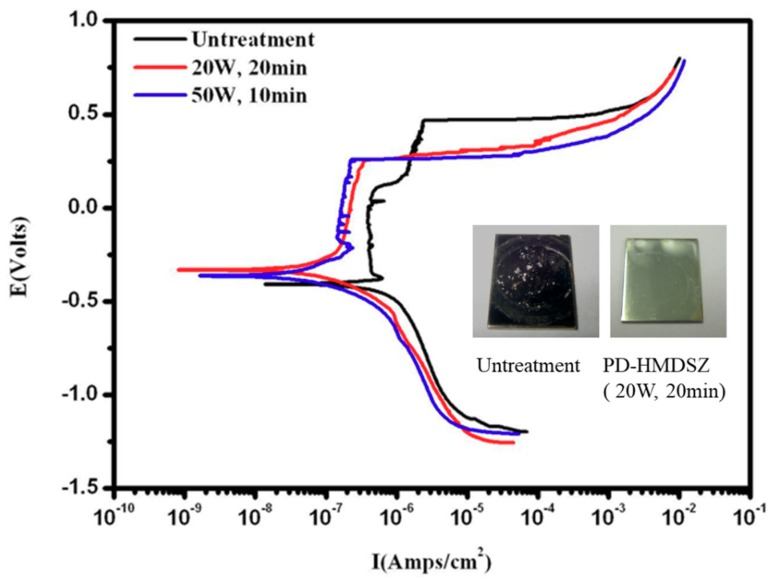
The polarization curves of HMDSZ-deposited stainless steel specimens under different plasma treatment conditions.

Figure 7 shows the polarization curves of HMDSZ-deposited stainless steel specimens under different plasma treatment conditions. Table 3 and Table 4 show the corrosion potential (Vssc) and current density (A/cm^2^) data of HMDSZ plasma deposited (20 W and 50 W, respectively, at 60 mTorr) onto stainless steel surfaces with different plasma treatment times. This figure indicates that after the treatment, the corrosion potential of the specimen increases and the corrosion current density decreases; therefore, the corrosion resistance is improved. The CCD camera shows the surface morphology of stainless steel via a potential dynamic test. The pitting holes were found on the stainless steel surface. The HMDSZ plasma deposited at 20 W and 60 mTorr for 20 min onto the stainless steel surface had fewer pitting holes then the others. Therefore, the plasma-modified film had better corrosion resistance interface than the others. In this study, the stainless steel modified by HMDSZ at 20 W and 60 mTorr for 20 min showed the best corrosion resistance with a corrosion current density of 1.0 × 10^−7^ decreasing to 1.0 × 10^−9^ A/cm^2^, and a corrosion potential of −0.121 increasing to 0.192 Vssc.

### 3.6. Thermo-Sensitive and pH-Sensitive Test of Composite Hydrogels

The weight of dry copolymer hydrogels (*W*_0_) was first measured. Next, the dry copolymer hydrogels were placed in solutions of PBS with pH = 4, 7.4 and 10 at 20 °C for 2 days to allow the copolymer hydrogels to reach an equilibrium swelling state. Following the setting at temperature of 25 °C, 28 °C, 31 °C, 34 °C, 37 °C, 40 °C, 45 °C, 50 °C, and 55 °C, respectively, the weight (*W*_1_) of each of the copolymer hydrogels was measured, and the swelling ratio (*SR*) was calculated using the formula:

*SR* = [(*W*_1_ − *W*_0_)/*W*_0_] × 100% (1)

Table 5 shows the variations of *SR* (%) for different hydrogels tested from 28 °C to 37 °C. The data in the table show that the composite hydrogels containing NIPAAm and 1 mol % AAc graft exhibit thermo-sensitive properties. Regardless of the pH value of PBS, the swelling ratio decreases by more than 200% when the temperature is increased from 28 °C to 37 °C. As the AAc concentration in the composite hydrogels is increased to 10 mol %, the thermo-sensitive phenomenon was insignificant. For the pH-sensitive test of composite hydrogels, Table 6 lists the variations of Δ*SR* (%) for different hydrogels tested from a pH = 4 to 10. As indicated in this table, the composite hydrogels containing 3 mol % AAc, 5 mol % AAc, and 10 mol % AAc show pH-sensitive properties. The swelling ratios at both temperatures were found to increase by more than 100% when the pH value changes from 4 to 10. When the AAc concentration is 5 mol %, and at 37 °C of PBS, the *SR* value reaches a maximum.

### 3.7. Drug Delivery Result

**Figure 8:**
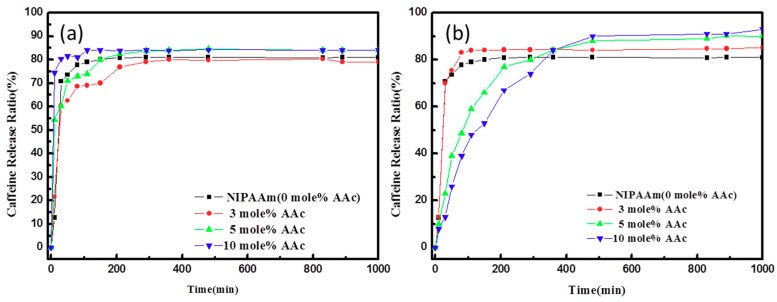
The caffeine drug release curves of different composite hydrogels tested at a temperature of 37°C of PBS and at (**a**) pH = 4, (**b**) pH = 10.

A caffeine drug delivery experiment was conducted on different composite hydrogels at a temperature of 37 °C of PBS. Figure 8 shows the caffeine drug release curves of different composite hydrogels tested at (a) pH = 4 and (b) pH = 10. From the swelling ratio measurement in the last section, it is implied that PNIPAAm is a thermo-sensitive hydrogel and AAc is an anionic pH-sensitive hydrogel. Comparing the result in Figure 8a with that of Figure 6b, it is known that at pH = 4, the drug delivery behavior of the thermo-/pH-sensitive composite hydrogels causes a volume contraction in response to a stress effect rather than by a diffusion mechanism, and at pH = 10, the reverse is true. Further, the drug delivery rate of the thermo-/pH-sensitive composite hydrogels in an acidic environment is faster than in an alkaline environment, but the drug delivery ratio in an acidic environment is smaller than that in an alkaline environment.

## 4. Conclusions

This study showed that in the plasma treatment with HMDSZ, the water contact angle of the treated specimen seems to change little with the treatment time, and short time HMDSZ plasma treatment is preferable to form a hydrophobic protected film. The stainless steel modified by HMDSZ at 20 W and 60 mTorr for 20 min showed the best corrosion resistance with a corrosion current density at 1.0 × 10^−9^ A/cm^2^ and corrosion potential at 0.192 Vssc. From the FTIR spectra and ESCA composition analysis, it was proven that HMDSZ and Poly (NIPAAm/AAc) composite hydrogels could be immobilized on the specimen surface. The swelling ratios of the thermo-/pH-sensitive composite hydrogels at a lower temperature and in an alkaline environment are larger than those at a higher temperature and in an acidic environment. The caffeine drug delivery experiment results revealed that the drug delivery rate of the thermo-/pH-sensitive composite hydrogels in an acidic environment is faster than an alkaline environment, but the drug delivery ratio in an acidic environment is smaller than that in an alkaline environment (release ratio up to about 90% of the absorbed amounts of caffeine after 8 h).

## Figures and Tables

**Table 1:** Feed composition of Poly (NIPAAm/AAc) composite hydrogels.

Gel Type	NIPAAm (mmol)	AAc (mmol)	APS (mmol)	NMBA (mmol)	TEMED (mmol)
**NIPAAm**	9.3	0.0	0.1	0.5	0.1
**1 mol % AAc**	9.2	0.1	0.1	0.5	0.1
**3 mol % AAc**	9.0	0.3	0.1	0.5	0.1
**5 mol % AAc**	8.8	0.5	0.1	0.5	0.1
**10 mol % AAc**	8.3	1.0	0.1	0.5	0.1

NIPAAm: *N*-isopropylacrylamide; AAc: acrylic acid; APS: ammonium persulfate; NMBA: *N*’-methylene-bis-acrylamide; TEMED: *N*,*N*,*N*′,*N*′-tetramethylethylenediamine.

**Table 2:** Atomic concentration of Poly (NIPAAm/AAc) composite hydrogels grafted onto the HMDSZ-treated substrate. Values obtained by ESCA.

Gel Types	Composition (at%)		
NIPAAm	C1_S_	O1_S_	N1_S_
0 mol % AAc	72.49	16.06	11.45
1 mol % AAc	66.3	22.34	11.36
3 mol % AAc	62.02	26.86	11.12
5 mol % AAc	72.57	18.77	8.66
10 mol % AAc	70.16	18.11	11.72

**Table 3:** Corrosion potential (Vssc) and current density (A/cm^2^) of PD-HMDSZ (20 W, 60 mtorr) onto a stainless steel surface with different treatment times.

	Potential (Vssc)	Current Density (A/cm^2^)
Untreatment	−0.121	1.0 × 10^−7^
3 min	−0.197	1.0 × 10^−8^
10 min	−0.054	1.0 × 10^−8^
20 min	−0.248	1.0 × 10^−7^
30 min	−0.104	1.0 × 10^−6^

**Table 4:** Corrosion potential (Vssc) and current density (A/cm^2^) of PD-HMDSZ (50 W, 60 mtorr) onto a stainless steel surface with different treatment times.

	Potential (Vssc)	Current Density (A/cm^2^)
Untreatment	−0.121	1.0 × 10^−7^
3 min	−0.026	1.0 × 10^−8^
10 min	0.087	1.0 × 10^−8^
20 min	0.192	1.0 × 10^−9^
30 min	−0.002	1.0 × 10^−9^

**Table 5:** Variations of *SR* (%) for different hydrogels tested from 28 °C to 37 °C.

pH	NIPAAm (mol % AAc)				
	0	1	3	5	10
4	218	208	170	161	8
7.4	218	204	17	33	16
10	218	202	46	34	56

**Table 6:** Variations of Δ*SR* (%) for different hydrogels tested at pH = 4, 7.4 and 10.

Temperature (°C)	NIPAAm (0 mol % AAc)				
	0	1	3	5	10
25	0	13	157	175	141
37	0	19	297	316	117

## References

[1] Park B.R., Nabae Y., Surapati M., Hayakawa T., Kakimoto M. (2013). Poly(*N*-isopropylamide)-Modified Silica Beads with Hyperbranched Polysiloxysilane for Three-Dimensional Cell. Polym. J..

[2] Small M., Faglie A., Craig A.J., Pieper M., Fernand Narcisse V.E., Neuenschwander P.F., Chou S.F. (2018). Nanostructure-Enabled and Macromolecule-Grafted Surfaces for Biomedical Applications. Micromachines.

[3] Choi S.H., Yoon J.J., Park T.G. (2002). Galactosylated Poly (*N*-isopropylacrylamide) Hydrogel Submicrometer Particles for Specific Cellular Uptake within Hepatocytes. J. Colloid Interface Sci..

[4] Chen K.S., Chou C.Y., Liao S.C., Wu H.M., Tsai H.T. (2012). Temperature Sensitivity of Composite Hydrogel Prepared by Surface Graft Polymer of NIPAAm and Bamboo Charcoal Powder. Biomed. Eng. Appl. Basis Commun..

[5] Chen M.A., Serizawa T., Li M., Wu C., Akashi M. (2003). Thermosensitive Behavior of Poly(*N*-isopropylacrylamide) Grafted Polystyrene Nanoparticles. Polym. J..

[6] Hsu S.H., Chen K.S., Lin H.R., Chang S.R. (2008). Effect of Plasma Gas Flow Direction on Hydrophilicity of Polymer by Small Zone Cold Plasma Treatment. Synth. React. Inorg. Metal. Org. Nano-Metal. Chem..

[7] Chou C.Y., Chen K.S., Lin W.L., Ye Y.C., Liao S.C. (2017). Plasma Polymerization of SnOxCy Organic-Like Films and Grafted PNIPAAm Composite Hydrogel with Nanogold Particles for Promotion of Thermal Resistive Properties. Micromachines.

[8] Chen K.S., Hung T.S., Wu H.M., Wu J.Y., Lin M.T., Feng C.K. (2010). Preparation Thermosensitive Gold Nanoparticles by Plasma Pretreatment and UV Grafted Polymerization. Thin Solid Films.

[9] Orhan M., Kut D., Gunesoglu C. (2012). Improving the Antibacterial Property of Polyethylene Terephthalate by Cold Plasma Treatment. Plasma Chem. Plasma Process..

[10] Osenberg F., Theirich D., Decker A., Engemann J. (1999). Process Control of a Plasma Treatment of Wool by Plasma Diagnostics. Surf. Coat. Technol..

[11] Kwon I.C., Bae Y.H., Kim S.W. (1991). Electrically Erodible Polymer Gel for Controlled Release of Drugs. Nature.

[12] Dusek K., Patterson D. (1968). Transition in Swollen Polymer Networks Induced by Intramolecular Condensation. J. Polym. Sci. A-2 Polym. Phys..

[13] Janca J., Sodomka L. (1998). Plasma-Polymerised Organosiloxane Thin Films as Selective Gas Sensors. Surf. Coat. Technol..

[14] Inagaki N. (1984). Preparation of Quaternary Nitrogen-Containing Plasma Films and Their Application to Moisture Sensors. Thin Solid Films.

[15] Inagaki N., Kishi A. (1984). Quaternary Nitrogen-Containing Polymer Films Prepared by Glow Discharge Polymerization. J. Polym. Sci. B Polym. Lett..

[16] Inagaki N., Kondo S., Hirata M., Urushibata H. (1985). Plasma Polymerization of Organosilicon Compounds. J. Appl. Polym. Sci..

[17] Zhang X.Z., Yang Y.Y., Wang F.J., Chung T.S. (2002). Thermosensitive Poly(*N*-isopropylacrylamide-co-acrylic acid) Hydrogels with Expanded Network Structures and Improved Oscillating Swelling-Deswelling Properties. Langmuir.

[18] Taylor D.K., Jayes F.L., House A.J., Ochieng M.O. (2011). Temperature-Responsive Biocompatible Copolymers Incorporating Hyperbranched Polyglycerols for Adjustable Functionality. J. Funct. Biomater..

[19] Lanzalaco S., Armelin E. (2017). Poly(*N*-isopropylacrylamide) and Copolymers: A Review on Recent Progresses in Biomedical Applications. Gels.

